# Modeling Tumor: Lymphatic Interactions in Lymphatic Metastasis of Triple Negative Breast Cancer

**DOI:** 10.3390/cancers13236044

**Published:** 2021-11-30

**Authors:** Kyungmin Ji, Zhiguo Zhao, Mansoureh Sameni, Kamiar Moin, Yong Xu, Robert J. Gillies, Bonnie F. Sloane, Raymond R. Mattingly

**Affiliations:** 1 Department of Pharmacology, Wayne State University, Detroit, MI 48201, USA; kji@med.wayne.edu (K.J.); msameni@med.wayne.edu (M.S.); kmoin@wayne.edu (K.M.); 2 Department of Electrical and Computer Engineering, Wayne State University, Detroit, MI 48202, USA; zhao.zhiguo@wayne.edu (Z.Z.); yongxu@wayne.edu (Y.X.); 3 Department of Cancer Physiology, Moffitt Cancer Center, Tampa, FL 33612, USA; robert.gillies@moffitt.org

**Keywords:** lymphatic metastasis, triple-negative breast cancer (TNBC), lymphatic endothelial cells (LECs), 3D cocultures, microfluidic chambers

## Abstract

**Simple Summary:**

Lymphatic metastasis is a critical prognostic factor of breast cancer aggressiveness and patient survival. Since existing therapeutic approaches have shown limited efficacy, new strategies to identify effective therapeutic targets for reducing breast cancer lymphatic metastasis are needed. We have used novel culture chambers, designed and fabricated by our group, to develop 3D models in which we can study spat ial interactions between breast cancer cells and lymphatic cells as they occur in real-time. This approach provides information on the complex cell–cell interactions involved in lymphatic metastasis of breast cancers. Factors in the secretome of the lymphatic cells promote invasive outgrowths from 3D cultures of breast cancer cells, suggesting that targeting interactions between breast cancer cells and lymphatic cells could be a potential therapeutic approach for the prevention of lymphatic metastasis.

**Abstract:**

Breast cancer frequently metastasizes to lymphatics and the presence of breast cancer cells in regional lymph nodes is an important prognostic factor. Delineating the mechanisms by which breast cancer cells disseminate and spatiotemporal aspects of interactions between breast cancer cells and lymphatics is needed to design new therapies to prevent lymphatic metastases. As triple-negative breast cancer (TNBC) has a high incidence of lymphatic metastasis, we used a three-dimensional (3D) coculture model of human TNBC cells and human microvascular lymphatic endothelial cells (LECs) to analyze TNBC:LEC interactions. Non-invasive analyses such as live-cell imaging in real-time and collection of conditioned media for secretomic analysis were facilitated by our novel microfluidic chambers. The volumes of 3D structures formed in TNBC:LEC cocultures are greater than that of 3D structures formed by either LEC or TNBC monocultures. Over 4 days of culture there is an increase in multicellular invasive outgrowths from TNBC spheroids and an association of TNBC spheroids with LEC networks. The increase in invasive phenotype also occurred when TNBC spheroids were cultured in LEC-conditioned media and in wells linked to ones containing LEC networks. Our results suggest that modeling spatiotemporal interactions between TNBC and LECs may reveal paracrine signaling that could be targeted to reduce lymphatic metastasis.

## 1. Introduction

### 1.1. Lymphatic Metastasis of Breast Cancer (BC)

Metastasis is the leading cause of mortality of BC patients [1,2] and is believed to initially occur through lymphatics, as would be consistent with the presence of BC cells in regional lymph nodes being an important prognostic indicator [3,4,5,6]. Nonetheless, surgical removal or irradiation of regional lymph nodes does not increase the survival of patients with early-stage breast cancer [7,8,9,10]. Whether regional lymph node metastases contribute to distant metastases cannot be directly assessed in patients; however, two recent studies in murine BC models have definitively established that BC cells do migrate from regional lymph node metastases through blood vessels to distant sites [11,12]. Lymphatic metastasis comprises both dissemination of BC cells to pre-existing lymphatics [13] and BC-induced lymphangiogenesis, thus bringing lymphatic vessels into the proximity of the tumor [6,14,15,16,17]. The two components are mediated at least in part by paracrine cytokine signaling between the BC cells and lymphatic endothelial cells (LECs) as verified by targeting single chemokines in murine BC models [18,19,20,21,22].

### 1.2. Lymphatic Metastasis of Triple Negative Breast Cancer (TNBC) 

TNBC accounts for 15–20% of all breast cancers and is characterized by the lack of expression of estrogen and progesterone receptors and of HER2 receptor amplification [23,24]. This BC subtype is associated with advanced stage at diagnosis, high rates of recurrence and poor outcome [23,25,26,27]. TNBC exhibits a high incidence of lymphatic metastasis (i.e., ~43%) [28,29] and extensive lymphangiogenesis, even in the absence of lymph node metastases [30]. Effective targeted treatments are urgently needed for TNBC, including ones directed toward the lymphatic metastasis of TNBC. 

### 1.3. Avatars for Studying Lymphatic Metastasis

Models in which cells are grown as 3D structures within extracellular matrices [31,32,33,34] have been found to better recapitulate the phenotype of those cells within an organism in regard to normal developmental as well as pathobiological processes [35,36,37,38,39,40,41,42,43,44]. 3D tumor models have been found to predict efficacy of a wide variety of cancer therapies [37,45,46,47] as well as replicate resistance to cytotoxic therapies [48,49,50,51,52]. Although drug discovery has long relied on 2D cultures and animal models for preclinical studies, 3D cultures have emerged as powerful new preclinical models that increase pathobiological relevance and successful translation to the clinic (see reviews [35,53,54]). Vascular metastasis has been modeled in 3D in microfluidic systems [55,56,57,58,59,60,61]. In contrast, there are few microfluidic systems for lymphatic metastasis. Those reported model transmigration of breast cancer cells into engineered tubules formed by monolayers of lymphatic endothelial cells [62,63,64]. 

Our group has developed and optimized 3D self-assembling BC cultures that can grow for extended periods of time (4D) and that support live-cell imaging, and molecular, biochemical and immunochemical analyses [65]. We have used such 3D cultures to study interactions between BC cells and other cells of the tumor microenvironment, e.g., fibroblasts, myoepithelial cells, macrophages [65,66,67,68,69,70]. We propose that 3D/4D cocultures will provide a significant and pathobiologically relevant tool for studying the underlying components of lymphatic metastasis in vitro. By performing spatiotemporal analyses of BC dissemination to LEC networks and of BC-induced lymphangiogenesis, we aim to identify therapeutic approaches for sequential targeting of multiple paracrine pathways that are likely to be more effective than targeting of single cytokine pathways or simultaneous targeting of multiple pathways have been. 

**Figure 1:**
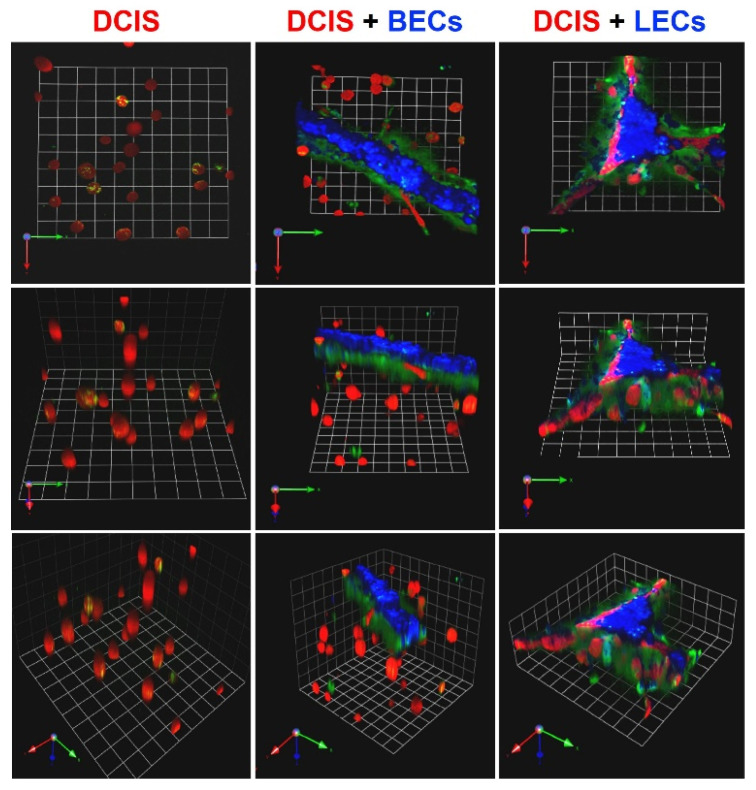
Ductal carcinoma in situ (DCIS) spheroids preferentially associate with microvascular lymphatic endothelial cell (LECs) networks. In 3D rBM overlay cultures, TN pre-invasive MCF10.DCIS cells form spheroidal structures; primary BECs form tubular structures; and primary LECs form triangular prism-like structures. Representative images of 3D reconstructions are shown en face and angled to illustrate the 3D structures formed by MCF10.DCIS-lenti-RFP (DCIS, red) cells alone (**left**) or in coculture with blood vessel microvascular endothelial cells (BECs, blue; **middle**) or LECs (blue; **right**). The rBM is mixed with DQ-collagen IV and green fluorescence represents degradation products of collagen IV. Cultures were imaged live at day 2; each grid represents 45 μm.

We have had a longstanding interest in using in vitro models to study BC progression, in particular the transition of ductal carcinoma in situ (DCIS) to invasive ductal carcinoma (for review [71]). To this end, we used 3D models consisting of a TN DCIS cell line, MCF10.DCIS, alone or in coculture with BC-associated cells. These studies demonstrated the ability of carcinoma-associated fibroblasts and myoepithelial cells to increase or decrease, respectively, the invasive transition of DCIS [69]. We have also developed 3D coculture models in which to analyze interactions of MCF10.DCIS with endothelial cells of lymphatic (LEC) and blood vessel (BEC) origin (Figure 1 and Supplemental Movies S1 and S2). To mimic metastasis as it occurs in vivo, the endothelial cells that we used were microvascular. Live-cell imaging of these 3D models revealed that MCF10.DCIS cells form spheroidal structures, BECs form tubular structures and LECs form triangular prism-like structures resembling lymph nodes (Figure 1). In cocultures, MCF10.DCIS spheroids are found in close association with the 3D structures formed by LECs yet not with those formed by BECs. These observations would be consistent with paracrine pathways in the TN DCIS:LEC cocultures that mediate dissemination of TN DCIS preferentially to lymphatics. Here we will describe the coculture models we have developed to identify pathways involved in lymphatic metastasis of TNBC and screen for new therapeutic approaches and the novel culture chambers that we designed to support non-invasive analyses of dynamic and spatiotemporal processes between TNBC spheroids and LEC networks as they self-assemble and interact in real-time.

## 2. Materials and Methods

### 2.1. Reagents

Reduced growth factor reconstituted basement membrane (rBM; Cultrex) was purchased from Trevigen (Gaithersburg, MD, USA). All culture media were phenol red-free, including Dulbecco’s Modified Eagle Medium (DMEM), mammary epithelial growth medium (MEGM), endothelial cell growth medium (EGM), and MycoZapTM Plus-CL, which were purchased from Lonza (Basel, Switzerland). Hyclone fetal bovine serum was from Cytiva (Marlborough, MA, USA). CellTrackerTM Orange, CellTraceTM Green, CellTraceTM Violet, and Kwik-Diff (methylene blue-eosin) stain kit were purchased from ThermoFisher Scientific (Waltham, MA, USA). DMEM/F12, horse serum, and all other chemicals, unless otherwise stated, were purchased from Sigma (St. Louis, MO, USA). Cignal Lenti-RFP was purchased from Qiagen (Hilden, Germany). Lentiviral vector pLenti-hTERT was purchased from Applied Biological Materials (Richmond, BC, Canada). A 3.7% formaldehyde solution was from Polysciences (Warrington, PA, USA). MycoFluor staining and LookOut RT-PCR kits for mycoplasma detection were purchased from Thermo Fisher Scientific (Waltham, MA, USA) and Sigma, respectively. BioCoat™ Control Cell Culture Inserts were purchased from Corning Life Sciences (Corning, NY, USA). Scratch- and UV-resistant cast polymethyl methacrylate (PMMA) sheets (black and transparent) were purchased from McMaster-Carr (Elmhurst, IL, USA). Medical-grade silicone was purchased from Weld-On Adhesives (Compton, CA, USA). 

### 2.2. Cells and Cell Maintenance 

MDA-MB-231 and Hs578T were purchased from ATCC and cultured in DMEM (Lonza) supplemented with 10% fetal bovine serum and 1% Mycozap Plus-CL. MCF10 human breast epithelial cell lines (MCF10.DCIS and MCF10.CA1d) were obtained from the Biobanking and Correlative Sciences Core of the Karmanos Cancer Institute (Detroit, MI, USA). MCF10.DCIS and MCF10.CA1d were maintained in DMEM/F12 supplemented with 5% horse serum. Human dermal microvascular LECs were immortalized with human telomerase reverse transcriptase (hTERT). One LEC line was a kind gift of Dr. Marion Groeger, University of Vienna, Austria and is designated LECs^MG^. These cells are characterized by strong expression of podoplanin, LYVE-1, and Flt-4/VEGFR-3 [72]. LECs purchased from Clonetics^TM^ Lonza were immortalized in our laboratory and are designated LECs^BFS^. All LECs were maintained in EGM. All cell lines were maintained in 2D monolayer cultures in phenol red-free culture media in a humidified incubator (5% CO_2_) at 37 °C. All cell lines were authenticated through the genotyping service of the Karmanos Biobanking and Correlative Sciences Core. In addition, routine testing by both staining and RT-PCR ensured that they remain free of mycoplasma contamination. 

### 2.3. 3D Culture 

**Figure 2:**
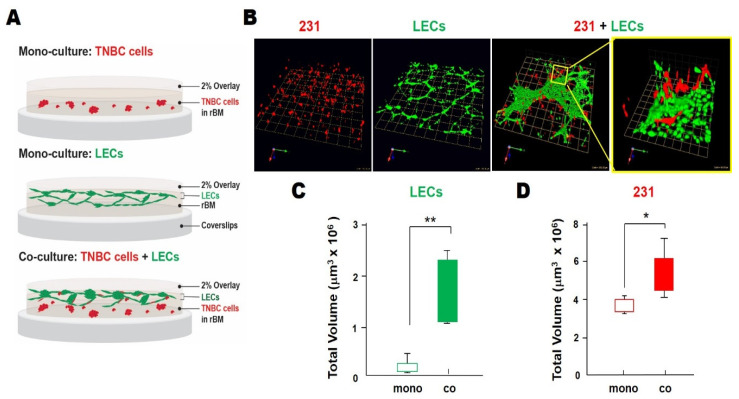
Increases in volumes of LEC networks and spheroids formed by the MDA-MB-231 TNBC cell line were observed when the two cell types were grown for 4 days in direct 3D coculture. (**A**) Schematics of monocultures and cocultures. (**B**) Representative images of 3D reconstructions of monocultures of 231s or LECs and 231:LEC^MG^ cocultures. Images are tiled from 16 contiguous fields; each grid represents 243 μm. Enlarged image on the right illustrates interactions between TNBC spheroids and LEC networks in greater detail; each grid represents 60 μm. (**C**,**D**) Total LEC and 231 volumes when grown alone (mono) or in direct coculture (co) were quantified with Volocity (*n* = 3). * *p*  ≤  0.05 and ** *p*  ≤  0.01.

We used a rBM overlay model optimized by our group for live-cell imaging by confocal microscopy [65,73]. For 3D cultures on glass coverslips, in monocultures of TNBC cells (8 × 10^3^ cells) or LECs (4 × 10^4^ cells), cells were seeded on 100% rBM (10 mg/mL rBM) on 12-mm diameter glass coverslips, overlaid with 1:1 mixture of MEGM and EGM culture media in 2% rBM (Figure 2A), and grown for 1 to 12 days. In 3D TNBC:LEC cocultures, TNBC cells were embedded in 100% rBM, LECs plated on top at a ratio of 1 TNBC cell to 5 LECs, and overlaid with 2% rBM diluted in a 1:1 mixture of MEGM and EGM culture media. For 3D cultures in TAME chambers, in monocultures and parallel cocultures of TNBC cells (8 × 10^4^ cells) or LECs^MG^ (4 × 10^5^ cells), cells were suspended in 15 μL of culture media and seeded on 100% rBM in the center of each well using TAME chambers with separate or linked wells. TNBC cells and LECs were labeled with CellTracker Orange and CellTrace Green or Violet, respectively, prior to seeding. MCF10.DCIS cells were transduced with Cignal Lenti-RFP to distinguish the DCIS cells from other cell types in 3D cocultures.

### 2.4. Image Acquisition for Quantitative Analysis in 3D

Optical sections of 16 contiguous fields or a single field through the entire depth of the 3D structures were acquired using a Zeiss LSM 780 or 410 confocal microscope (Carl Zeiss Microscopy, Jena, Germany), respectively. Image stitching of contiguous fields was done in x–y dimensions with Zen Black software (Carl Zeiss, Jena, Germany). For z-stacks, images were reconstructed in 3D using Volocity software (PerkinElmer, Waltham, MA, USA). Volumes of total structures, invasive outgrowths, and cores of 3D structures were quantified using Volocity software as previously described [70]. X (green), y (red), and z (blue) arrows in the bottom left of each image indicate orientation of 3D images.

The interaction/overlap between 231 spheroids and LEC networks was quantified in 3D reconstructions of 231:LEC^MG^ cocultures using MATLAB image processing and segmentation modules in a hierarchical step method (see Supplemental Figure S2). First, 3D images were processed with contrast enhancement, image smoothing, noise removal and segmentation, then masks of LEC and TNBC structures were created (Supplemental Figure S2A). Next, the segmented images were used to decide which TNBC spheroids were inside LEC networks and which were outside (Supplemental Figure S2B). To quantify the interaction between LECs and TNBC, we computed the volume of the TNBC spheroids inside LEC networks and divided by the total volume of the LEC networks. 

### 2.5. LEC-Conditioned Media (CM)

LECs^MG^ (1 × 10^6^ cells in a 60 mm dish) in fresh EGM, i.e., EGM not exposed to cells, in 2% rBM were added on top of 100% rBM and maintained for 4 days. Media were collected, centrifuged at 1800× *g* to remove debris and floating cells, and supernatants aliquoted to avoid damage from multiple freeze–thaw cycles. The resulting conditioned media from LECs (LEC-CM) or the cell-free condition (control media; CtrlM) were stored at −80 °C until use. A total of 231 cells (8 × 10^3^ cells) were then seeded on 100% rBM, overlaid with 2% rBM and grown in the presence of either CtrlM or LEC-CM mixed at a ratio of 1:1 with fresh MEGM; media were replaced every other day. 

Heat-inactivated LEC-CM were prepared by boiling for 15 min at 100 °C. Freeze/thaw (F/T) LEC-CM were generated by subjecting LEC-CM to 3 consecutive cycles of 15 min at −80 °C followed by 15 min at 60 °C and centrifugation at 1800× *g* to remove cells and cell debris [74]. LEC-CM were fractionated using Amicon centriprep centrifugal ultrafiltration units with a 3 kDa cutoff at 4000× *g* for 50 min (Beckman L7-65; Indianapolis, IN, USA), according to the manufacturer’s protocol (MilliporeSigma, Burlington, MA, USA). The retentate (molecules larger than 3 kDa; >3 kDa) and eluate (molecules smaller than 3 kDa; <3 kDa) were collected and stored at −80 °C until use. Control media or LEC-CM subjected to boiling, repeated F/T, or size exclusion cut-off were mixed at a ratio of 1:1 with fresh MEGM; media were replaced every other day.

### 2.6. 3D Invasion Assays

Invasion assays were performed as we have previously described [67]. Briefly, 0.8 μm BioCoat control filter inserts were coated with 50 μL of 5 mg/mL rBM. A total of 231 cells (3 × 10^3^ cells) in MEGM were seeded and cultured on rBM-coated inserts in 24-well plates for 5 days to allow formation of 231 spheroids and then incubated for a further 2 days with either control media (CtrlM) or LEC^MG^-CM in the lower compartment. Non-invasive cells were removed from the top of the inserts with a cotton swab. Cells on the bottom of the inserts, i.e., that had invaded, were fixed with 3.7% formaldehyde and stained with Kwik-Diff (methylene blue & eosin). After the stained filters were mounted on slides, invaded cells were visualized using a Zeiss Axioplot-200 microscope (Carl Zeiss, Jena, Germany) and quantified using ImageJ software (NIH). Images of five random fields per insert were acquired and two independent experiments were carried out in triplicate. 

### 2.7. Fabrication of TAME Chambers

We made PMMA-based TAME chambers by CO_2_ laser machining (EpilogLaser, Golden, CO, USA). The current chamber prototype consists of 12 separate wells or 6 pairs of linked wells. TAME chambers consist of three layers of PMMA sheets. The lid (6 mm thickness) made of transparent PMMA contains inlet/outlet gas flow channels (2 mm in diameter) distributed to each well. The bottom is 2 layers of black PMMA bonded with medical-grade silicone (Weld-On Adhesives, Compton, CA, USA). The lower layer (6 mm thickness) contains culture wells, inlet/outlet ports (2 mm in diameter) for delivery/removal of media, and a microfluidic integrated gradient generator. This is covered by a thin film (1 mm thickness). Each cylindrical culture well in the bottom layer has a size equivalent to that of a well in a conventional 24-well culture plate. Each culture well is perfused through connection to a peristaltic pump; reservoirs of medium are connected to the inlets; and outlets are connected to tubing that is used to withdraw media for collection of CM. The peristaltic pump precisely controls the rate and direction of media flow. The integrated gradient generator is a Christmas tree-like microfluidic network [75] that has 2 inlets (2 mm in diameter) to introduce two different drugs or reagents. Microfluidic network patterns for the integrated gradient generator were first patterned using a laser scan speed of 15% and power of 30%, with the laser set to low power raster mode, creating channels of ~2 mm in depth. Culture wells were created with a scan speed of 15% and power of 70%, with the laser set to a high-power vector mode. Gas inlets/outlets in the lid were drilled using a mechanical drill bit and media inlets/outlets in the bottom were formed by sealing the grooves. The design (e.g., sizes and numbers of culture wells, inclusion of integrated gradient generators) can be easily modified.

### 2.8. Statistical Analysis

Data are presented in box-and-whisker plots or line graphs. In box-and-whisker plots, boxes represent interquartile ranges and whiskers minimum and maximum values. Time-course data are presented as line-graphs. The significance of differences was evaluated by one-way ANOVA, followed by Tukey’s post hoc test to correct for multiple comparisons. For all studies, *p* ≤ 0.05 was considered statistically significant.

## 3. Results

### 3.1. TNBC:LEC Interactions in 3D Cocultures

**Figure 3:**
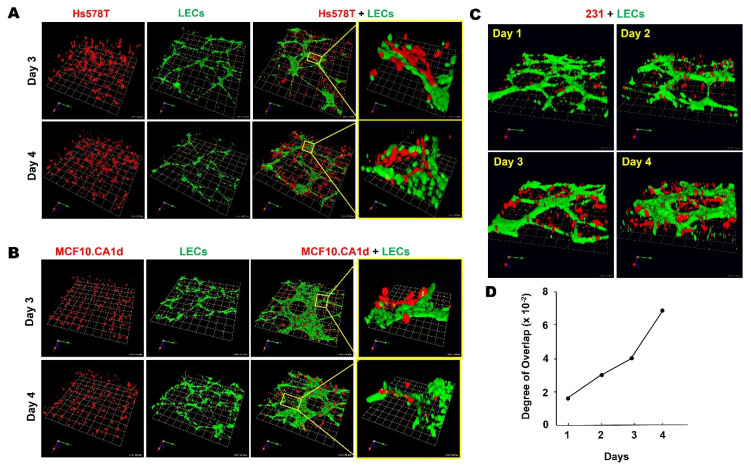
Spatiotemporal changes in interactions between TNBC spheroids and LEC networks could be observed over 4 days of direct 3D coculture. (**A**,**B**) Representative images of 3D reconstructions of monocultures of Hs578T (**A**), MCF10.CA1d (**B**) or LECs^BFS^ (**A**,**B**) and Hs578T:LEC (**A**) or MCF10.CA1d:LEC (**B**) cocultures on days 3 and 4. Images are tiled from 16 contiguous fields; each grid represents 243 μm. Enlarged images on the right illustrate interactions between TNBC spheroids and LEC networks in greater detail; each grid represents 43 μm. (**C**) Representative time-course images of 3D reconstructions of 231:LEC^MG^ direct cocultures. Images are tiled from 16 contiguous fields; each grid represents 243 μm. (**D**) Quantitative analysis demonstrated a time-dependent increase in 231:LEC overlap over the 4 day period in direct 3D coculture. Degree of overlap = volume of 231s inside LEC networks/total volume of LEC networks.

We assessed over a 4-day period the self-assembly and growth of 3D structures in rBM overlay cultures by human TNBC cells and human microvascular LECs. We compared monocultures in which the two cell types were grown separately and direct cocultures in which they were grown together in the same well (Figure 2A). Consistent with findings by Bissell and colleagues [76,77], the MDA-MB-231 TNBC cell line (hereafter called 231) formed spheroidal structures that over time in culture exhibited multicellular invasive outgrowths. In contrast, hTERT-immortalized LECs^MG^ (Univ. Vienna) [72] formed branching networks with central nodes linked in a reticular pattern (Figure 2B), mimicking the growth patterns of lymphatics in vivo [78]. In cocultures, LEC^MG^ networks were substantially larger, and 231 spheroids were found primarily in association with LEC^MG^ networks (Figure 2B). Total volumes of both LEC^MG^ networks and 231 spheroids (Figure 2C,D) were significantly greater in the cocultures than in the monocultures, suggesting that paracrine interactions enhance TNBC growth and LEC growth/remodeling. To confirm that comparable results would be obtained with other LECs, we purchased LECs from Clonetics^TM^ Lonza and immortalized them with hTERT in our laboratory, i.e., LECs^BFS^. We observed similar spatiotemporal changes in TNBC: LEC^BFS^ interactions over a 4-day period of with two other TNBC cell lines, Hs578T (Figure 3A) and MCF10.CA1d (Figure 3B), as well as with a preinvasive MCF10.DCIS TN cell line (Supplemental Figure S1). A time-dependent increase in 231:LEC^MG^ overlap (Figure 3C,D) was verified by quantitative analysis, using MATLAB software to measure the degree of overlap in reconstructed 3D confocal images that had been processed and segmented into 231 spheroids and LEC^MG^ networks (Supplemental Figure S2).

### 3.2. LEC Secretome and TNBC Invasion

**Figure 4:**
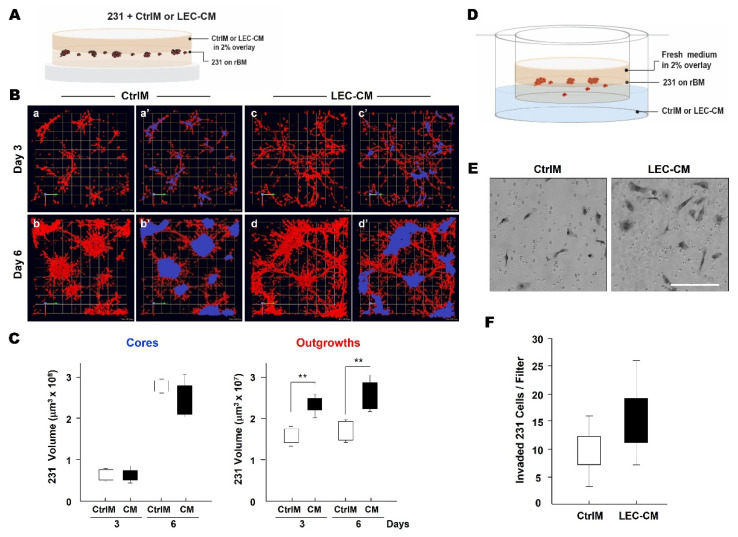
LEC secretome increases invasive phenotype of MDA-MB-231 spheroids. (**A**) Schematic illustrating protocol for incubation of 231 spheroids in 3D rBM overlay cultures with control media (CtrlM) or LEC^MG^-conditioned media (LEC-CM or CM). (**B**) En face views of representative 3D reconstructions of 231 spheroids (red) in CtrlM (a,b) or LEC-CM (c,d) at 3 and 6 days. To quantify effects on invasive phenotype, 231 spheroids were segmented as illustrated in a’–d’ into central cores (blue) and multicellular invasive outgrowths (red) using Volocity software. Images are tiled from 16 contiguous fields; each grid represents 250 µm. (**C**) Volumes of 231 cores and multicellular invasive outgrowths at days 3 and 6 of incubation with CtrlM or LEC-CM (CM) were quantified with Volocity as previously described [70]; ** *p*  ≤  0.01. (**D**) Schematic of in vitro 3D invasion assay: 231 spheroids were cultured in the presence of control media (CtrlM) or LEC^MG^-conditioned media (LEC-CM). (**E**) DIC (differential interference contrast) images of invaded cells attached to the underside of the insert. Scale bar, 200 μm. (**F**) Invasion of 231 cells from spheroids incubated in the presence of CtrlM or LEC-CM for 2 days was quantified with ImageJ.

The observed time-dependent increase in association of TNBC spheroids with LEC networks in 3D cocultures suggests that paracrine factors secreted from the LEC networks might mediate TNBC dissemination to lymph nodes. We therefore examined whether LEC secretome has any effect on the invasive phenotype of TNBC spheroids. Effects of LEC^MG^-conditioned media (CM) or control media that had not been exposed to cells (CtrlM) were compared at 3 and 6 days of incubation (Figure 4A). The 231 cells grew into stellate spheroidal structures when cultured in CtrlM. When cultured in LEC^MG^-CM the 231 spheroids exhibited a more invasive phenotype in which spheroids were linked by multicellular invasive outgrowths between spheroids (Figure 4B). Changes in the volume of the invasive outgrowths were quantified in 3D reconstructed images that had been segmented into central cores (Cores) and invasive outgrowths (Outgrowths). Core volume, which is indicative of cell proliferation, was not affected by exposure to LEC^MG^-CM, but did increase 3- to 4-fold between 3 and 6 days in culture (Figure 4C). In contrast, the invasive phenotype of 231 spheroids was substantially affected by exposure to LEC^MG^-CM. The volume of the multicellular invasive outgrowths significantly increased at both 3 and 6 days of culture (Figure 4C). To determine whether the observed increases in invasive outgrowths corresponded to increases in cell invasion, we used a Boyden chamber assay that we modified to assess invasion of cells from 231 spheroids [67] rather than from monolayers of 231 cells, i.e., 3D invasion (Figure 4D). Invasion was increased in the presence of LEC-CM as a chemoattractant in the lower compartment (Figure 4E,F). Our results are consistent with a paracrine factor in the LEC-CM (LEC secretome) that stimulates TNBC invasion.

### 3.3. Tissue Architecture and Microenvironment Engineering (TAME) Chambers

We encountered a few problems in using commercial culture chambers for 3D/4D cultures. To maintain these cultures over the time required to self-assemble into 3D structures, we seed the cells on a layer of 100% rBM and overlay the cells with 2% rBM. Unfortunately, the rBM blocks inlet and outlet ports through which media are perfused, limiting the viability of the 3D structures. Many commercial culture chambers are not suitable for live-cell imaging by confocal microscopy and live-cell assays, non-disruptive addition or removal of therapeutic agents or collection of CM, analysis of dose–response effects, etc. Therefore, we designed our own culture chambers, i.e., TAME chambers (Patent: US 10,227,556 B2). The TAME chambers are fabricated of PMMA since it is durable, inexpensive, and non-porous [79]. Furthermore, PMMA does not offgas endocrine disruptors or absorb small, hydrophobic molecules such as estrogen unlike the more commonly used PDMS (polydimethylsiloxane). PDMS is thus of particular concern for studies of BC, over extended periods in culture and for testing effects of small, hydrophobic molecules [35].

**Figure 5:**
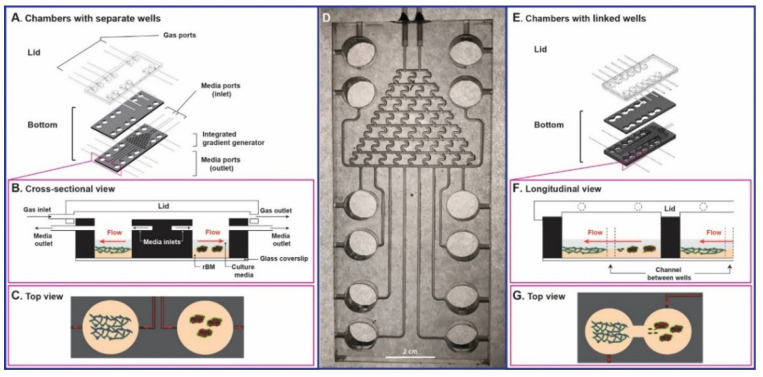
Schematics of TAME chambers with separate wells (**A**–**D**) or linked wells (**E**–**G**). Exploded (**A**,**E**), cross-sectional (**B**), longitudinal (**F**), and top (**C**,**G**) views of chambers with separate (**A**–**C**) or linked (**E**–**G**) wells. (**D**) Photograph of prototype of chamber with separate wells and an integrated gradient generator.

Figure 5A–C illustrates TAME chambers in which the culture wells are separate, allowing one to grow monocultures of single cell types in individual wells or direct cocultures in which multiple cell types are grown in contact with one another in individual wells. A prototype of this design, fabricated with transparent PMMA to facilitate testing of the integrated gradient generator, is shown in Figure 5D. Less complex TAME chambers with 4 separate wells were used in the present studies for static culture. Figure 5E–G illustrate linked-well chambers in which each pair of wells is linked so that a monoculture of one cell type in one well can be linked to a monoculture of another cell type in the second well. Thus, the linked-well chambers support parallel cocultures in which the secretome from one cell type is shared with that of the other cell type and are ideal for evaluating the effects of media flow from one cell type to the other, including the effects of reversing flow. The bottom two layers of TAME chambers are routinely made with black PMMA to reduce autofluorescence and optical glass slides are used to cover the bottom of each pair of wells, facilitating live-cell imaging at high resolution on an inverted confocal microscope. 

### 3.4. LEC Secretome-Induced TNBC Invasion in TAME Linked-Well Chambers

**Figure 6:**
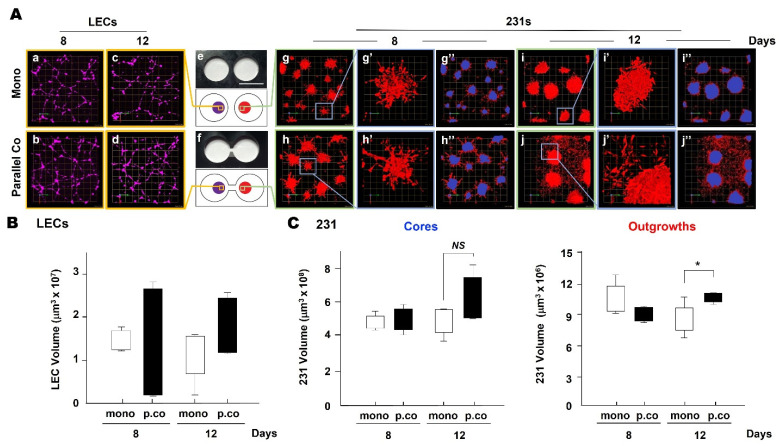
Multicellular invasive outgrowths from 231 spheroids are induced over a 12-day period in parallel 3D cocultures of 231 spheroids and LEC networks in TAME linked-well chambers. (**A**) Photos (top e, f) and schematics (bottom e, f) of TAME chambers with separate (top row) or linked (bottom row) wells. En face views of 3D reconstructions of 3D monocultures (Mono) in separate-well chambers (top row) and parallel 3D cocultures (Parallel Co) in linked-well chambers (bottom row) of 231 s (red) and LECs^MG^ (purple) on days 8 and 12. Images are a single field with each grid in a–d, g–j, and g”–j” representing 50 μm. Enlarged images (g’–j’) illustrate TNBC spheroids in greater detail; each grid represents 10 μm. Scale bar in e represents 1.6 cm. (**B**) Total LEC volumes when grown alone (mono) or in parallel coculture (p.co) with 231 s were quantified at days 8 and 12 with Volocity (*n* = 3). (**C**) Volumes of central cores and invasive outgrowths formed by 231 cells in 3D monoculture (mono) or parallel 3D coculture (p.co) with LECs at days 8 and 12 were quantified with Volocity as previously described [70]. * *p*  ≤  0.05; NS, not significant.

We grew 3D monocultures of LECs^MG^ and 231s in parallel coculture in TAME chambers with linked wells. In this configuration, media are shared between 231 spheroids and LEC networks, yet there is not direct contact between the two cell types. We compared invasive phenotypes of 231s grown in monoculture in separate wells to those of 231s grown in parallel coculture in linked wells (Figure 6 and Supplemental Figure S3). Networks formed in 3D by LECs^MG^ were similar at 8 and 12 days of culture in the two configurations (Figure 6A). Significant differences in total LEC volume were not observed, yet there was somewhat more variability in total LEC volume at both days 8 and 12 in the parallel coculture configuration (Figure 6B). Phenotypes of 231 spheroids grown in 3D in monoculture or parallel coculture in linked wells were similar at days 1 and 4 (Supplemental Figure S3) and 8 (Figure 6A). At day 12, 231 spheroids grown in parallel coculture in linked wells exhibited an enhanced invasive phenotype in which both multicellular outgrowths and individual cells were observed migrating out of the 231 spheroids (Figure 6A). We used segmentation to discriminate cores from invasive outgrowths and quantified changes in volume of cores and outgrowths. We did not observe significant changes in core volume. In contrast, the volume of invasive outgrowths from 231 spheroids was significantly greater at 12 days of parallel coculture (Figure 6C). These findings in parallel 3D cocultures resemble those observed when 231 spheroids in monoculture were incubated with LEC-CM (cf., Figure 4C and Figure 6C). In both cases, the volumes of invasive outgrowths from 231 spheroids were increased. The increase in invasive phenotype in parallel cocultures was observed at 12 days whereas that in monocultures treated with LEC-CM was observed already at 3 days. This may reflect the time required in the parallel coculture configuration for a paracrine factor(s) to move from the LEC network in one linked well to the 231 spheroids in the other linked well before inducing a response. Our results are consistent in supporting the LEC secretome inducing an increase in the invasive phenotype of TNBC spheroids. 

**Figure 7:**
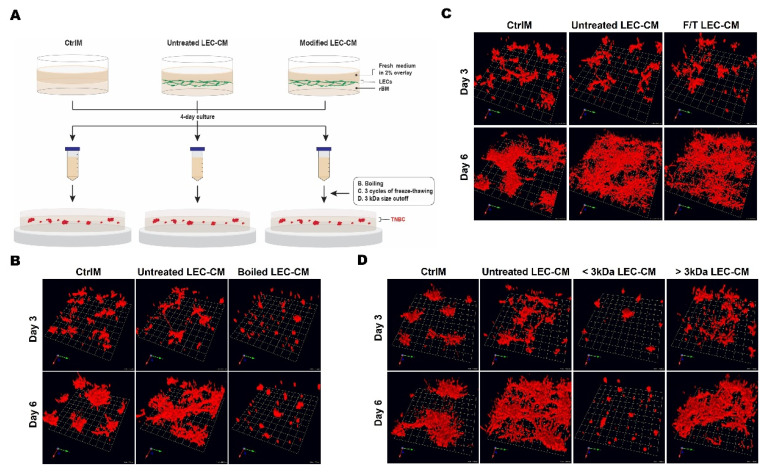
Effects of LEC secretome on increases in invasive outgrowths of 231 spheroids are retained following repeated freeze/thaw (F/T) cycles, but abolished by boiling, or by centrifugal ultrafiltration using filters for molecules less than 3 kDa in size. (**A**) Schematic illustrating protocol for incubation of 231 spheroids in 3D rBM overlay cultures with untreated or boiled, F/T, or 3 kDa cutoff LEC^MG^-conditioned media. (**B**–**D**) Angled images of 3D reconstructions of 231 spheroid monocultures (red) grown for 3 or 6 days in CtrlM, untreated LEC-CM, or LEC-CM subjected to boiling, 3 consecutive cycles of freezing and thawing, or centrifugal ultrafiltration using a 3 kDa cutoff filter. Images are tiled from 16 contiguous fields; each grid represents 170 μm.

Initial analyses of LEC-CM were conducted to characterize the nature of the paracrine pathways that mediate increases in invasiveness of TNBC spheroids. We tested the effects of subjecting LEC-CM to boiling (100 °C, 15 min), three consecutive freeze–thawing (F/T) cycles (−80 °C, 60 °C), or centrifugal ultrafiltration with a 3 kDa size cut-off filter (Figure 7A). We first tested the thermal stability of candidate molecules to boiling. The invasive phenotype of 231 spheroids was increased by incubation with untreated LEC^MG^-CM as compared to that of 231 spheroids incubated with CtrlM (Figure 7B); also see Figure 4B,C. In contrast, 231 spheroids incubated with boiled LEC^MG^-CM were smaller and less invasive than those incubated with either CtrlM or untreated LEC^MG^-CM (Figure 7B). These results are consistent with boiling having inactivated a paracrine factor(s) that mediates invasiveness and a growth factor(s) that mediates proliferation. Repeated F/T cycles damage secondary, tertiary, and quaternary structures of proteins [80]. LEC^MG^-CM subjected to three F/T cycles retained its ability to induce invasive outgrowths from 231 spheroids (Figure 7C). LEC^MG^-CM retentate and eluate, fractionated using centrifugal ultrafiltration units with a 3-kDa cutoff filter, were tested for their ability to induce an invasive phenotype in 231 spheroids. The retentate containing paracrine factors larger than 3 kDa retained the ability to induce invasive outgrowths from 231 spheroids, whereas the eluate containing paracrine factors smaller than 3 kDa did not support development of an invasive phenotype nor did it support growth of the spheroids (Figure 7D). These results are consistent with TNBC invasiveness being mediated by paracrine pathways involving proteins > 3 kDa in size.

## 4. Discussion

We present evidence that 3D culture models grown in TAME chambers can be employed to follow self-assembly of TNBC spheroids and LEC networks and spatiotemporal interactions between the spheroids and networks by live-cell imaging. Two types of TAME chambers are described here. One with separate wells supports monocultures of TNBC spheroids or LEC networks or TNBC spheroids and LEC networks growing in direct contact in the same wells. The other with linked-wells supports parallel cocultures of TNBC spheroids and LEC networks growing without direct contact in separate wells linked by a channel. Sizes of culture wells and the positioning of inlet and outlet ports are optimized for present 3D coculture models but could be easily modified. The TAME chambers use a perfusion system for the introduction of media for growth and long-term maintenance of cells and for collection of CM non-invasively for secretomic analysis at either specific times or continuously. The TAME chambers are designed for use as drug screening platforms. They are constructed from PMMA, a thermoplastic, rather than the more commonly used PDMS. PDMS releases endocrine disruptors, which could be of concern over long times in culture and have been shown to transactivate the estrogen receptor in an ovarian carcinoma cell line stably transfected with an estrogen receptor element and a luciferase transporter gene [81]. PDMS also absorbs small hydrophobic molecules [82,83] and thus is not suitable for dose–response testing of drugs, whereas thermoplastics are [82,83]. Beebe and colleagues conclude in a recent review that there is a need to transition away from proof-of-concept in PDMS devices to devices made of materials that are applicable to drug testing [84]. Our TAME chambers can be fabricated with an integrated gradient generator with branching microchannel networks, which will reproducibly generate a gradient of drug concentrations for dose–response studies. This is connected to a peristaltic pump to maintain flow rates of drug introduction and withdrawal and avoid undesirable shear stress on the cultures. 

The TAME chambers provide a standardized and reproducible platform for growing 3D BC and LEC models, which undergo self-assembly into BC spheroids and LEC networks. Both the BC spheroids and the LEC networks replicate the 3D structures observed in vivo [76,77,78]. In contrast, others have modeled lymphatic metastasis of BC by engineering assembly of LEC monolayers on the exterior of a PDMS rod [64], a hydrogel block in a PDMS device [63], or a FluoroBlok culture insert [62]; lymphatic metastasis of BC spheroids was not analyzed in those studies. We have established that the TAME chambers support: (1) self-assembly of TNBC cells into spheroids and LECs into networks without introducing a spatially-constrained artificial or engineered architecture; (2) growth and maintenance of 3D cultures/cocultures of TNBC spheroids and LEC networks over extended periods to allow analysis of spatiotemporal interactions between 3D structures; (3) monitoring and quantification of dynamic, spatiotemporal changes in phenotype of 3D cultures/cocultures by live-cell imaging protocols; (4) temporal and non-invasive collection of CM (secretome) without disturbing the cultures/cocultures; (5) direct coculture of TNBC spheroids and LEC networks in TAME chambers with separate wells; (6) parallel coculture of TNBC spheroids and LEC networks in TAME chambers with linked wells; and (7) culture of TNBC spheroids in LEC-CM in TAME chambers with separate wells. In short, the TAME system (TAME chambers + 3D cultures/cocultures) allows one to analyze either direct or paracrine interactions in 3D as they occur in real-time under control or experimental conditions. Here we used the TAME system to demonstrate recruitment of TNBC spheroids to LEC networks when they were grown in direct contact or were linked through paracrine pathways and spatial and temporal effects on TNBC:LEC interactions. 

Lymphatic metastasis is complex and involves tumor-induced lymphangiogenesis and dissemination of tumor cells to pre-existing lymphatics, suggesting that effective therapies will need to target both lymphangiogenesis and tumor dissemination. The TAME system described here can model lymphangiogenesis, tumor dissemination, and the reciprocal paracrine interactions between tumor cells and LECs that are being explored as therapeutic targets in a variety of different tumors (for reviews on lymphatic metastasis, see [6,17,85]). Targeting a single paracrine signaling pathway, e.g., CCL21/CCR7 [22] or CXCL12/CXCR4 [18], has been shown to reduce lymphatic metastasis in murine models of breast cancer. A multi-component pathway linked to lymphatic metastasis of TNBC has been identified in vivo in a murine model and in vitro in a 2D culture model [86]. Interleukin 6 is secreted by TNBC cells, inducing LECs to secrete CCL5, and in turn recruiting TNBC cells to lymphatics. Targeting this paracrine loop between LECs and TNBC cells reduces lymphatic metastasis. These findings are consistent with the complex, spatiotemporal processes we observed in the TAME system as LEC networks/LEC secretome induced an increase in multicellular invasive outgrowths from TNBC spheroids. Our TAME system studies provide a template for mechanistic analyses to precisely define the spatial and temporal aspects of this process, identify druggable targets, and incorporate this spatial and temporal information into design and testing of therapies. 

## 5. Conclusions

We have found that the LEC secretome plays a critical role in increasing the invasive phenotype of TNBC spheroids using self-assembling 3D culture models grown and analyzed in our novel TAME chambers. We also demonstrated the applicability of our novel TAME chambers to studies of paracrine interactions over time between two cell types growing in 3D, in this case between TNBC spheroids and LEC networks. The TAME chambers facilitate non-invasive analyses by live-cell imaging and secretome collection/analysis. This system should be applicable for screening therapeutic targets associated with lymphatic metastasis in other cancers as well as therapeutic targets associated with interactions between tumors and their microenvironment.

## 6. Patents

K.J., K.M., Y.X. and B.F.S. are co-inventors on a patent for the TAME chambers (Patent #, US 10,227,556 B2). 

## Data Availability

Data are available upon request from the corresponding author.

## Supplementary Materials

The following are available online at https://zenodo.org/record/5033236#.YaY_ztDMJPZ (DOI: 10.5281/zenodo.5033236), Supplemental Figure S1: Spatiotemporal changes in interactions between TN DCIS spheroids and LEC networks could be observed over 4 days of direct 3D coculture, Supplemental Figure S2: Image processing and segmentation for quantification of TNBC:LEC interaction/overlap, Supplemental Figure S3: Phenotypes of LEC networks and 231 spheroids in monoculture or in parallel coculture in TAME linked-well chambers at 1 or 4 days, Supplemental Movie S1: Video of MCF10.DCIS spheroids interacting with BEC 3D tubule-like structures, Supplemental Movie S2: Video of MCF10.DCIS spheroids interacting with LEC 3D lymph node-like structure.
